# Effects of emotional valence and arousal on acoustic duration reproduction assessed via the “dual klepsydra model”

**DOI:** 10.3389/fnbot.2014.00011

**Published:** 2014-02-28

**Authors:** Jiří Wackermann, Karin Meissner, Dharol Tankersley, Marc Wittmann

**Affiliations:** ^1^Institute for Frontier Areas of Psychology and Mental HealthFreiburg im Breisgau, Germany; ^2^Institute of Medical Psychology, Ludwig Maximilian UniversityMünchen, Germany; ^3^Department of Psychiatry, University of California San DiegoSan Diego, CA, USA

**Keywords:** arousal, dual klepsydra model, duration reproduction, emotion, time perception

## Abstract

We report results of an acoustic duration reproduction task with stimulus duration of 2, 4, and 6 s, using 45 emotionally negative, positive, and neutral sounds from the International Affective Digitized Sounds System, in a sample of 31 young healthy participants. To investigate the influence of induced emotions on perceived duration, the effects of emotional modulation were quantified in two ways: (1) via model-free indices (aggregated ratios of reproduced times), and (2) via dual klepsydra model (dkm)-based estimates of parameters of internal time representation. Both data-analytic approaches reveal an effect of emotional valence/arousal, namely, a significantly longer reproduction response for emotional stimuli than for the neutral stimuli. The advantage of the dkm-based approach is its ability to disentangle stimulus-related effects, which are represented by “flow intensities,” from general effects which are due to the lossy character of temporal integration. We explain the rationale of the dkm-based strategy and interpret the observed effect within the dkm-framework as transient increase of internal “flows.” This interpretation is in line with recent conceptualizations of an “embodiment” of time where the model-posited flows correspond to the ongoing stream of interoceptive (bodily) neural signals. Neurophysiological findings on correlations between the processing of body signals and the perception of time provide cumulative evidence for this working hypothesis.

## 1. Introduction

Perception of duration is known to be dependent on many factors, from physical characteristics of perceived events (Goldstone and Goldfarb, 1963; Block, 1978; Grondin, 1993) to psychophysiological states of the perceiving subject (Wittmann, 2009; Mella et al., 2011; Droit-Volet et al., 2013a). Experimental procedures and data-analytic models that allow us to disentangle and selectively test the role of different modulating factors are required for a better understanding of processes underlying time perception, and temporal consciousness in general.

In the present study we investigated the perception of temporal intervals marked by acoustic stimuli of varied emotional character, using the duration reproduction paradigm. The theoretical framework of the study was the hypothesis of bodily states being the physiological basis for time perception (Wittmann, 2009; Wittmann et al., 2010a). This hypothesis is based on the embodiment approach by Craig (2009) who proposes that our perception of time relates to emotional and visceral processes that all share a common underlying neural system within the interoceptive system and the insular cortex. In this context, several studies have shown how emotions and bodily arousal lead to an overestimation of duration. The experience of duration thereafter emerges from the processing and representation of emotional and body states (Droit-Volet and Gil, 2009; Droit-Volet et al., 2013b; Wittmann, 2013). This hypothesis fits conceptually with models of internal representation of temporal duration based on the integration of intra-organismic “flows” which may be tentatively identified with streams of ascending neural signals (Meissner and Wittmann, 2011; Sysoeva et al., 2011). Specifically, we use a “klepsydraic” model, belonging to the class of “lossy integration” models, to quantify, test and interpret observed effects.

**Figure 1:**
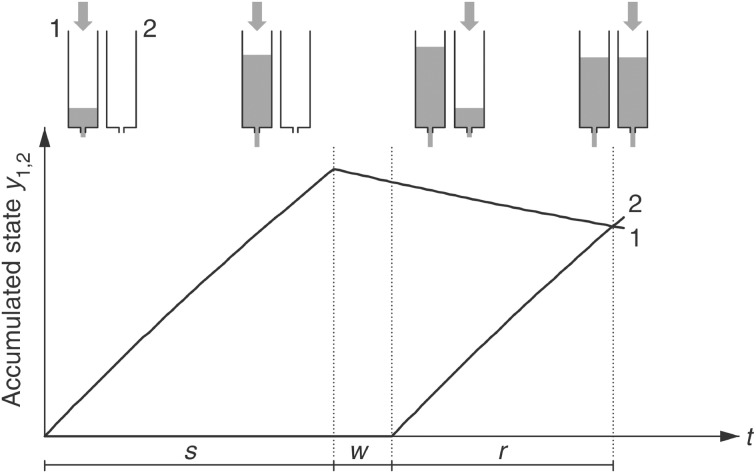
**Dual klepsydra model of duration reproduction**. *s* = encoded duration, *w* = inter-stimulus interval, *r* = reproduced duration. [From Wackermann and Ehm (2006) with permission from Elsevier Science.]

The “dual klepsydra model” (dkm) (Wackermann and Ehm, 2006) assumes that duration of an attended time interval is represented by the state of an inflow– outflow unit (iou), acting as a “lossy integrator” (see Appendix A for details). In the duration discrimination or reproduction task, two integrators are allocated, one being active (“filled”) during the first interval (“encoding” phase), the other during the second interval (comparison or reproduction phase). Importantly, neither the “inflows” entering the integrators nor the states of the integrators are observable; they thus do not provide a direct measure of “subjective time.” Only the result of their comparison is accessible to the subject's awareness. In a duration reproduction task, equality of the states of the two integrators is indicated by the subject's verbal or motor response (e.g., key press) according to the instruction (Figure 1). The integrators are subject to continuous outflow, at a rate proportional to their momentary states. Due to this “lossy” character of the ious, reproduced duration *r* is not a veridical reproduction but rather a non-linear function of “encoded” duration *s* [Appendix A, Equation A3]. The dkm thus naturally accounts for the progressive shortening of the relative reproduction response (i.e., decrease of the ratio *r*/*s* with increasing *s*), which is an ubiquitous phenomenon in duration reproduction experiments in the supra-second range (Späti, 2005; Wackermann and Ehm, 2006; Wackermann et al., 2008; Pütz et al., 2012).

The dkm shares the concept of integrator (sometimes called “accumulator”) with the “pacemaker–counter” model (pcm), which is influential in the time perception literature (Zakay and Block, 1997). In the dkm the input to integrators are continuous “flows,” while the pcm postulates series of discrete “pulses,” but this does not make a substantial difference: counting is only a special case of integration. The essential difference between the two models consists in their operating principles. In the dkm approach, different areas of the neural substrate may be allocated and re-used as integrators (cf. Jech et al., 2005), their states are (bio)physical states, and their magnitudes compared directly. By contrast, the pcm is based on an idea of a central “counter,” states of which are transferred to auxiliary “registers,” and from there compared numerically with memory data. Briefly, the dkm is a sort of primitive, analog computer implemented in the biological matter, while the pcm mimics digital computing machinery (Wackermann, 2012).

Yet another difference between the two models is that the dkm makes it possible to separate, conceptually as well as computationally, effects due to the lossy character of neural representation (outflow) from effects caused by variation of the integrator input (inflow). The PCM, in its classic form, could account for effects of all kind only in terms of variation of the effective pulse frequency—i.e., number of “pulses” incoming at the accumulator per (physical) time unit (Treisman, 1963)—unless the memory or decision components are conceived of as active structures, such as in variants of the Scalar Timing models where the representation of duration is modified (Gibbon et al., 1984; Wearden, 2004). In the dkm, the same unit serves as integrator and memory storage, and the internal representation results from two concurrent processes, inflow and outflow. This is important in the context of the present study, where we aimed at changes in subjectively perceived duration induced by experimental variations of the emotional valence of acoustic stimuli marking temporal intervals. To assess these effects, we have to disentangle them from the omnipresent “progressive shortening” effect mentioned above. In the dkm this is possible, because the two kinds of effects are represented by two model parameters, κ, regulating the outflow rate, and η, representing inflow ratios between the encoding and reproduction phase (Appendix A).

The aim of the present study was to explore the effects of emotional modulation on duration reproduction in the supra-second range (2–6 s), using acoustic stimuli from a standardized, valence and arousal level rated system (IADS; Bradley and Lang, 2007). So far only a few studies have employed affective sounds in the analyses of temporal processes using the IADS (Noulhiane et al., 2007; Mella et al., 2011). With this approach we aimed at extending evidence for the relationship between time perception and the processing of acoustic emotional content. In addition, we applied a data-analytic procedure based on the dual klepsydra model to differentiate valence/arousal-related effects from general, emotion-unspecific effects. With this strategy we present a novel parametric approach to analyze effects of varied stimulus quality on perceived duration.

## 2. Materials and methods

### 2.1. Participants

Thirty-one subjects (16 women), students from the local university between 19 and 34 years of age (mean = 25.2 years; *S.D*. = 3.2 years) were recruited for this study. All participants reported good health and no known neurological or psychiatric problems. They signed an informed consent before the beginning of the session and were reimbursed for taking part in the study.

### 2.2. Design and stimuli

The International Affective Digitized Sounds System (IADS-2, Bradley and Lang, 2007) consists of 167 naturally occurring sounds that are rated according to the emotion dimensions of “valence,” “arousal,” and “dominance.” Forty-five stimuli were selected from the IADS-2 and assigned to three groups of 15 sounds each, according to their valence rating

- Positive (p), e.g., pleasant music, a boy laughing, erotic sounds;
- Negative (n), e.g., babies crying, a person vomiting, an alarm clock ringing; and
- Neutral (e), e.g., clicking of type-writer, rain, animal noises.

The positive (pleasant) and negative (unpleasant) sounds were selected such that they were comparable regarding average arousal rating. The reference numbers of the stimuli and statistics of ratings for the stimulus groups are shown in Table 1.

**Table 1:** **Acoustic stimuli used in the study and their average ratings**.

**Group**	**IADS no**.	**Pleasantness**		**Arousal**	
		**Mean**	***SD***	**Mean**	***SD***
**POSITIVE**					
*a*	200, 254, 802, 817, 820	6.58		6.27	
*b*	110, 351, 366, 717, 810	7.22		5.78	
*c*	220, 311, 201, 355, 816	7.14		6.60	
Total		6.98	0.51	6.22	0.96
**NEGATIVE**					
*a*	252, 293, 600, 611, 709	3.33		5.96	
*b*	255, 288, 296, 712, 730	2.82		6.35	
*c*	250, 260, 282, 711, 732	3.29		6.51	
Total		3.15	0.59	6.27	0.98
**NEUTRAL**					
*a*	113, 246, 705, 720, 724	4.97		4.41	
*b*	102, 130, 376, 700, 701	4.87		4.55	
*c*	322, 361, 627, 722, 728	4.80		4.67	
Total		4.88	0.17	4.54	0.38

This relatively large amount of stimuli was chosen in order not to present a given stimulus twice to a subject. The experiment consisted of 45 trials = the number of selected sounds (15 of each group). Three different durations, 2, 4, and 6 s, were used in the duration reproduction task. This range of durations was chosen because the acoustical stimuli necessitate certain duration for being meaningful and evoking an emotion. Being too short they might not be deciphered correctly and when being too long, they might evoke modulations in subjective reactions. The stimuli have been validated for 5 s duration.

Accordingly, each of the three stimulus groups (positive, negative, neutral valence) was split into three sub-groups of five sounds each (*a*, *b*, *c*), which were assigned to the three duration conditions. The complete factorial structure of the experiment was thus 3 emotional valences (P, N, E) × 3 durations (*s* = 2,4,6) × 5 stimuli per sub-group.

### 2.3. Procedure

The experiment was controlled by a portable computer, using a program written in Matlab (Mathworks Inc., Natick, MA) in conjunction with the Psychophysics Toolbox extensions for PC (Brainard, 1997). Stimuli were presented via headphones (Sennheiser HD 201). Stimuli were presented to all subjects with the same loudness, preset at a comfortable hearing level.

Each trial started with the presentation of one of the IADS-2 stimuli for 2, 4, or 6 s (encoding phase). After a pause of either 1.5, 2, or 3 s, a pure sinewave tone of 150 Hz frequency was presented. Subjects had to stop the tone by pressing the space-bar when they felt that its duration had reached the duration of the first stimulus (reproduction phase). The next trial started after a variable inter-trial interval with durations of either 3, 3.5, or 4 s. Subjects received four practice trials before the proper experimental phase.

Since it is tempting for subjects to use chronometric counting as a strategy to be more accurate, participants were strongly requested not to count and not to use any other strategies (e.g., subvocal singing) to measure the duration of sounds. It was emphasized that we were not interested in their counting abilities but in their subjective impression of duration. We did not employ a secondary task as it may interfere with the actual timing task. It has been shown that merely instructing subjects not to count is the best strategy for assessing interval timing abilities (Rattat and Droit-Volet, 2012).

## 3. Data reduction and analysis

### 3.1. Data preprocessing

Following the procedure described above, data from 45 trials were obtained from each subject, with five trials for each combination of three emotional valences (*v* = N, P, E) and three different presented durations (*s_i_* = 2,4,6 s for *i* = 1,2,3, respectively). Time elapsed from the onset of the second tone to the space-bar press was measured as the subject's response *r_v,i,j_*. These data were reduced to arithmetic means,

(1) $${\bar{r}}_{v,i}=\frac{1}{5}\sum_{j=1}^{5}r_{v,i,j}$$

for further analyses. (To simplify the notation, the superscripted bar will be omitted in the following text. The symbol *r_v,i_* is to be understood as the average across a five-trial sub-group for given valence *v* and duration *s_i_*.)

In the subsequent sections we apply two parallel strategies in order to aggregate data over different durations *s_i_*. The first strategy is purely descriptive, representing effects of varied stimulus valence by means of simple arithmetic indices; the second strategy is based on parametrization of the effects via the dkm. In this way we separate a straightforward demonstration of the existence of expected effects (model-free analysis) from their more refined quantification and interpretation (model-based).

### 3.2. Aggregate ratio indices

To assess the effect of emotional valence *v* = n, p with respect to (w.r.t.) the neutral condition e, we calculated for each subject the mean ratio of reproduction responses

(2) $$a_{v}=\frac{1}{3}\sum_{i=1}^{3}\frac{r_{v,i}}{r_{\text{E},i}},$$

taking the average over all three durations. If stimulus valence has no effect on the response, the expected value of the ratios *r_v,i_*/*r*_E_,*i*, and therefore of the aggregated ratios *a_v_*, are 1 (Appendix B). This will be tested across the sample of 31 subjects using a one-sample *t*-test^1^.

### 3.3. DKM-based analysis

To assess the effect of emotional valence *v* = N, P w.r.t. to the neutral condition e, we estimate inflow ratios between the encoding and reproduction phase, and from these we calculate net inflow ratios between the emotionally laden and neutral stimuli. (See Equations 6, 7 in Appendix C, where also a detailed rationale for the analysis is given). For each individual subject, the procedure consists of the following steps:

1. Estimate κ from the merged data-set consisting of all 45 trials, assuming formally η = 1.
2. Using the estimate of κ from step 1, estimate η_*v*_ separately for *v* = P, N, E, each subset consisting of 15 trials.
3. Using the estimates from step 2, calculate ratios η_pe_ = η_p_/η_e_ and η_ne_ = η_n_/η_e_.

Similar to the model-free analysis described in the preceding subsection, the null-hypothesis-based expected value of inflow ratios η_pe_ and η_ne_ are 1. We proceed in the same vein, using one-sample *t*-tests to test for deviations from the expected value.

## 4. Results

### 4.1. Descriptive statistics

The grand means and standard deviations of reproduced duration calculated across all 31 subjects are summarized in Table 2. The statistics of reproduced duration are shown as functions of the encoded duration *s* = 2, 4, and 6 s, sorted by emotional conditions P (positive), N (negative) and E (neutral), and for all trials regardless of the emotional condition (the bottom row).

**Table 2:** **Statistics of reproduced durations (grand means and standard deviations) sorted by emotional valence and encoded duration *s***.

	***s* = 2 s**		***s* = 4 s**		***s* = 6 s**	
**Valence**	**Mean**	***SD***	**Mean**	***SD***	**Mean**	***SD***
Positive	2.335	0.690	3.717	0.869	4.799	1.102
Negative	2.136	0.571	3.456	0.876	4.839	1.106
Neutral	2.210	0.676	3.496	0.937	4.527	1.212
All	2.227	0.646	3.556	0.892	4.722	1.137

As a global test of significance a Two-Way ANOVA was computed, with factor 1 = Valence (three levels) and factor 2 = Duration (three levels). The results (Greenhouse–Geisser corrected) are:

- Factor Valence: *F* = 8.089 (*df* = 2,60); *P* < 0.001.
- Factor Duration: *F* = 308.075 (*df* = 2,60), *P* < 0.0001 (trivial).
- Interaction Valence versus Duration: *F* = 3.809 (*df* = 4,120); *P* = 0.012.

Calculating *post hoc t*-tests for dependent variables (*df* = 30) within duration conditions and between emotional conditions, we found

- For *s* = 4 s, a significant difference between conditions P and E (*t* = 2.600, *P* < 0.05);
- for *s* = 6 s, significant differences between conditions N and E (*t* = 2.986, *P* < 0.01), as well as between conditions P and E (*t* = 2.226, *P* < 0.05).

**Figure 2:**
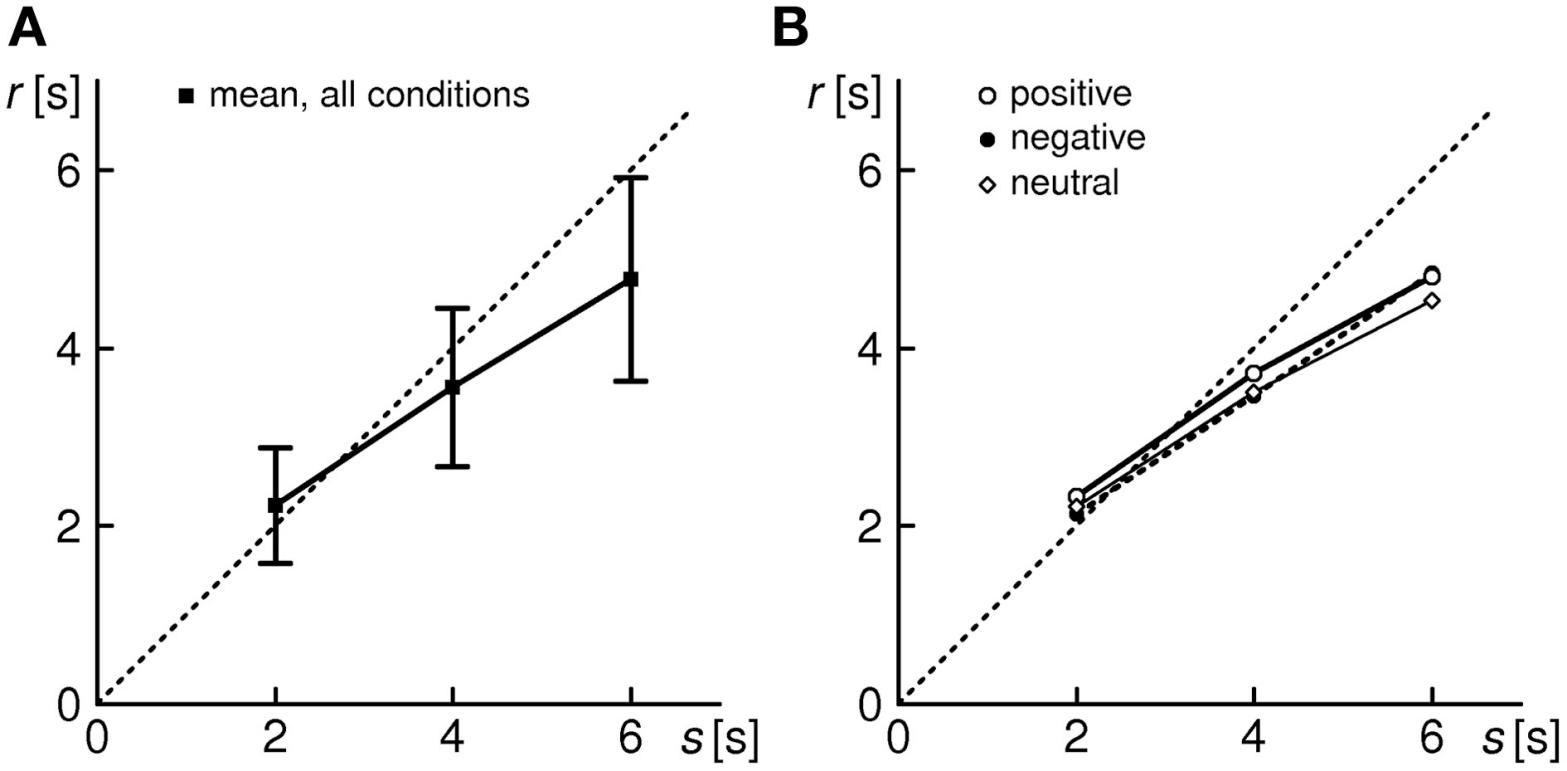
**Average reproduction response for the entire sample of 31 subjects**. **(A)** All conditions (shown are grand means ± 1 *SD*). **(B)** Separated by conditions e,n,p (*SD* bars omitted for the sake of legibility).

In addition, a review of the reproduction responses relatively to the encoded duration *s* reveals, on the average, a minor (non-significant) over-reproduction for *s* = 2 s, and a pronounced under-reproduction for *s* = 4 and 6 s (Figure 2). We thus observe the progressive shortening effect, consistent with other duration reproduction studies (cf. Introduction).

### 4.2. Aggregate ratio indices

**Figure 3:**
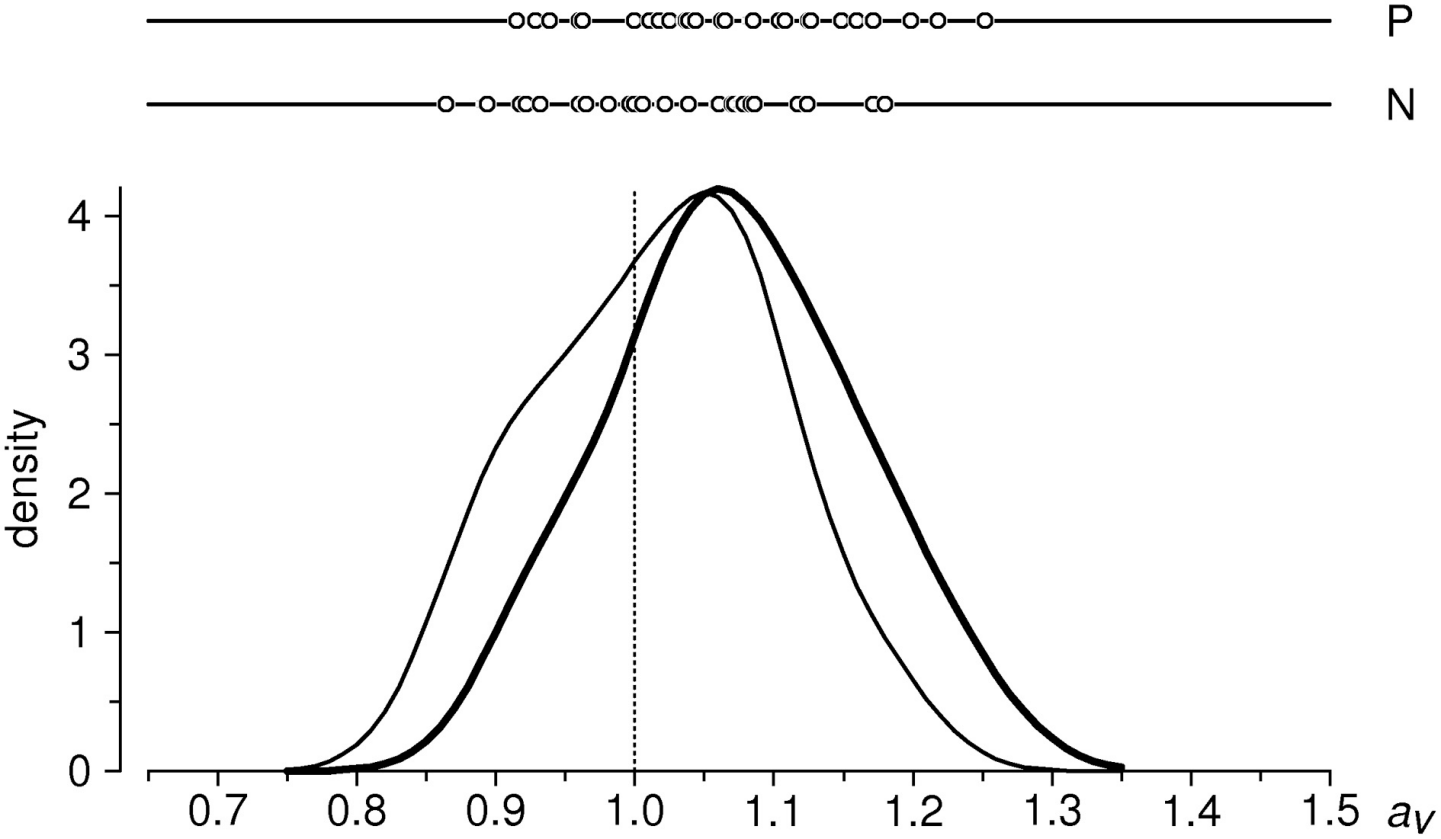
**Distributions of aggregated ratio indices *a_P_* and *a_N_* in the entire sample (*n* = 31 subjects**). Upper part: individual data points; below: estimates of probability density functions (thin curve: *a*_n_, thick curve: *a*_p_), obtained with a Gaussian kernel (Silverman, 1986) of bandwith σ = 0.04. Vertical dotted line indicates the null-hypothesis-based expectation.

Empirical distributions of *a*_n_, *a*_p_ in the sample of 31 subjects are shown in Figure 3; sample means are 1.0175 and 1.0715, respectively. One-sample *t*-test against the expected value of 1 yields *t* = 1.171 (ns) for the condition N, and *t* = 4.646 (*P* < 0.001) for the condition P (*df* = 30 for both tests). We thus have a highly significant lengthening effect of ~7% for positive valence stimuli; in addition, we observe a small (1.75%) but not significant difference in the same direction for negative valence stimuli^2^.

### 4.3. DKM-based analysis

Results of κ estimates based on krf fits to merged data-sets were a necessary pre-requisite for further analysis; therefore these results are reported first. In four cases we obtained an estimate of κ = 0. Data inspection revealed that these four participants consistently and sometimes vigorously over-reproduced the presented duration *s*. This kind of response, sometimes seen in a minority of subjects (cf. Pütz et al., 2012), cannot be accounted for by the dkm. A negative value of κ would be required to fit these data, but κ < 0 is biophysically meaningless; in such cases the estimation procedure stops at the lower-bound κ = 0.

Furthermore, for one subject we obtained an estimate of κ = 0.24 s^−1^. Visual inspection of her data revealed that her responses *r* varied in a very narrow range and only minimally reflected the *s* durations presented in the encoding phase. It is unclear whether she misunderstood the instruction or exhibited an unusual kind of cognitive bias. In any case, this extreme value, largely deviating from the rest of the sample, is to be considered an evident outlier.

The four over-reproducing subjects and the one non-responder were removed from the data-base; the cleaned sample thus consists of 26 subjects. In this sample, κ values were in the range from 0.003 to 0.096 s^−1^, median $\tilde{\kappa }$ = 0.03 s^−1^.

**Figure 4:**
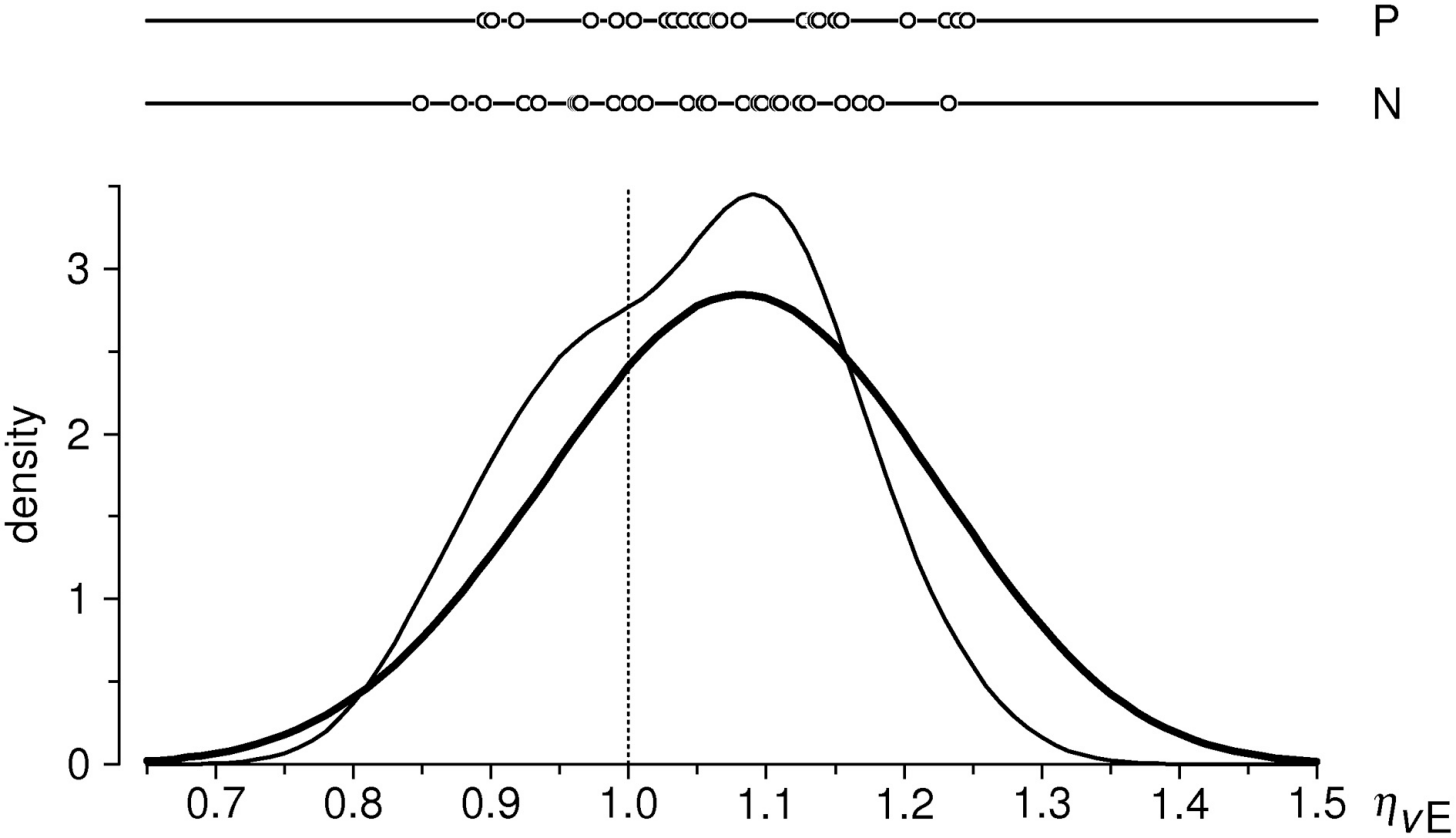
**Distributions of estimated flow ratios η_pe_ and η_ne_ in the cleaned sample (*n* = 26 subjects)**. Upper part: individual data points; below: estimates of probability density functions (thin curve: η_pe_, thick curve: η_ne_), obtained with the same method as in Figure 3. Vertical dotted line indicates the null-hypothesis-based expectation.

Empirical distributions of the inflow ratios η_ne_ and η_pe_ in the sample of 26 subjects are shown in Figure 4; sample means are 1.0421 and 1.0788, respectively. One-sample *t*-test against the expected value of 1 yields *t* = 2.133 (*P* < 0.05) for η_ne_, and *t* = 4.081 (*P* < 0.001) for η_pe_ (*df* = 25 for both tests). We thus observe increase of inflow ratios w.r.t. the neutral stimuli in both emotion conditions: a moderate (~4%) and significant effect for negative valence stimuli, and a large (~8%) and highly significant effect for positive valence stimuli^3^.

## 5. Discussion and conclusion

Our results complement earlier studies showing that emotional stimuli are judged to last longer than more neutral stimuli of the same physical duration (Droit-Volet and Gil, 2009; Wittmann, 2009). An overestimation of duration is more likely seen with negative emotional valence. However, the presentation of positive emotional stimuli can also lead to a relative overestimation of duration as compared to neutral stimuli (Lambrechts et al., 2011), results which our data supports. In fact, in our task with the specific selection of sounds from the IADS battery, positive sounds are relatively longer reproduced than negative sounds, both emotional stimuli being relatively over-estimated compared to neutral sounds. Such findings of an overestimation are typically discussed within the framework of an increased physiological arousal level that leads to a higher frequency of an internal pacemaker underlying time perception (Wittmann and Paulus, 2008; Gil and Droit-Volet, 2012). A higher speed of an internal clock would lead to the accumulation of more pulses emitted by a hypothetical pacemaker operating in the seconds range (Zakay and Block, 1997). For example, in one study a faster tempo in music, which was related to an increased subjective arousal, produced relative duration overestimates as compared to slow tempi (Droit-Volet et al., 2013b). In another study, temporal intervals were judged to last longer after subjects had viewed a frightening film that increased fear and arousal levels (Droit-Volet et al., 2011). In this line of evidence, looming stimuli that were virtually approaching the viewer were relatively overestimated as compared to steady stimuli or those that were virtually receding (Wittmann et al., 2010b).

In the present analytical approach we want to frame the results related to the temporal overestimation of emotionally arousing stimuli within a novel framework provided by the dual klepsydra model of duration representation. Wackermann and Ehm, (2006, p. 489) suggested that external stimulus-induced, phasic effects are mediated by changes in integrator inflows (assessed by parameter η), whereas effects of the global organismic state and its slow, tonic changes, are mediated by the outflow rate (assessed by parameter κ). There is now substantial empirical support for this conjecture. Parameter κ has been shown to be a test-retest stable, probably genetically co-determined, individual characteristic (Sysoeva et al., 2010). This does not mean that κ is perfectly constant; it is subject to intra-individual circadian variations (Späti, 2005), and can be affected by neurochemical agents acting on the synaptic level (Wackermann et al., 2008). All these findings point out to the slow, tonic character of κ reactivity, on time scales of hours or longer. On the other hand, the inflow ratio η has been shown to reflect physical properties of stimuli used in a time perception task (brightness–duration interaction; Wackermann and Meyer-Blankenburg, 2009). It is thus reasonable to expect that the inflow ratio η is the proper “locus of effect” also for stimulus-induced influences of other kinds.

The working hypothesis of the present study is based on the conjecture of two different loci of the effects discussed above. Stimuli of high emotional valence—and, usually of high biological relevance—induce a general physiological response (arousal), resulting in a temporary increase of interoceptive afferences. We hypothesize that the observed effects on time perception are due to a temporary increase of neural flows feeding the hypothetical integrators. Therefore, phasic changes of arousal level should be measurable in terms of inflow ratios between arousing versus emotionally neutral conditions. To obtain robust and valid estimates of these changes, we aggregate reproduction data over the entire range of encoded duration *s*. This is an important difference from the majority of duration reproduction studies testing effects for different values of *s* separately. In our approach it is the response function *r* = *f(s)* in its entirety that carries information on the internal duration representation. Accordingly, instead of seeking/testing effects in the data space, we first apply a mathematical model to the data and then test for the expected effects in the parameter space.

In the present paper we used two data-evaluation strategies in parallel, to prevent a possible criticism for moulding the data to our preferred model. The results of both strategies are well comparable: Pearson's correlation between *a*_n_ and η_ne_ is *R* = +0.857 (*df* = 24, *P* < 0.001), and between *a*_p_ and η_pe_ it is *R* = +0.878 (*df* = 24, *P* < 0.001). We can thus say that the aggregated ratio indices (model-free) and the inflow ratios (model-based) measure almost the same—yet not exactly the same thing. The dkm-based strategy turns out to be more sensitive, particularly w.r.t. effects induced by negative-valence stimuli, seemingly non-significant when using the ratio indices, but significant when quantified in terms of inflow ratios^4^. Arguably, this better sensitivity is due to separation of stimulus-specific effects from the unspecific effect of “progressive shortening.” The latter effect is demonstrated by the summary statistics of reproduction responses (Table 2), showing an average shortening of ~11 % for *s* = 4 s, and ~21 % for *s* = 6 s. This effect is further evidenced by the distribution of individual κ values which are positive for the great majority of subjects; the sample median value of $\tilde{\kappa }$ = 0.03 s^−1^ is in very good agreement with κ values found in earlier studies (Späti, 2005; Wackermann and Ehm, 2006; Wackermann and Späti, 2006; Wackermann et al., 2008; Sysoeva et al., 2010; Wittmann et al., 2011; Pütz et al., 2012).

The main finding of the present study is a lengthening of reproduction response for emotional stimuli w.r.t. to the response for neutral stimuli: we found significant effects for both conditions P and N versus E. The two dimensions are not independent in our selection of stimuli: both stimulus groups P and N show on the average higher arousal than the group E (cf. Table 1). Therefore we cannot clearly differentiate between influences of the two stimulus dimensions; nonetheless, our results are in line with the general arousal-increase hypothesis specified above.

**Figure 5:**
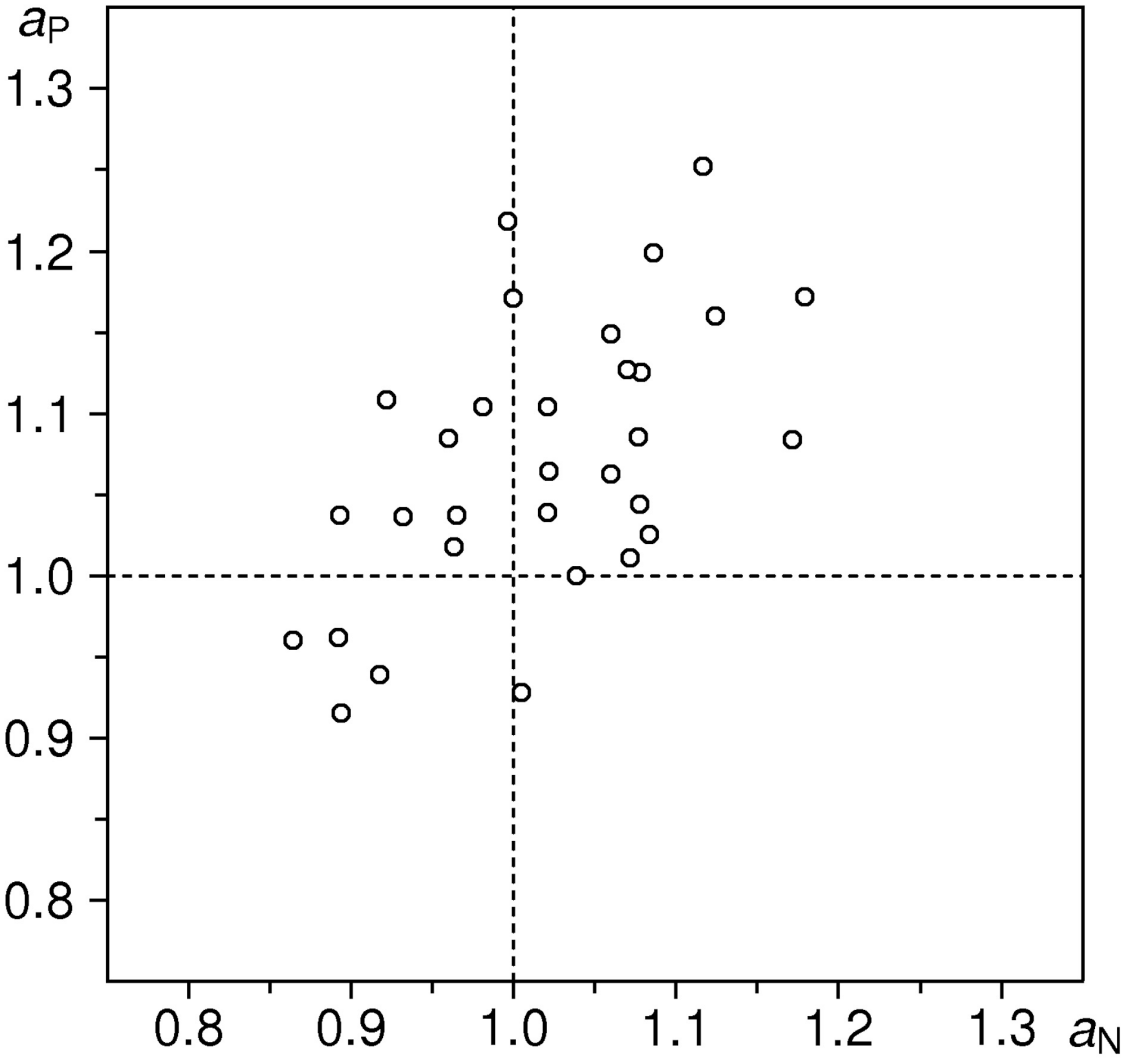
**Correlation plot of aggregated ratio indices *a*_n_ versus *a*_p_ (*n* = 31, *R* = +0.566)**.

**Figure 6:**
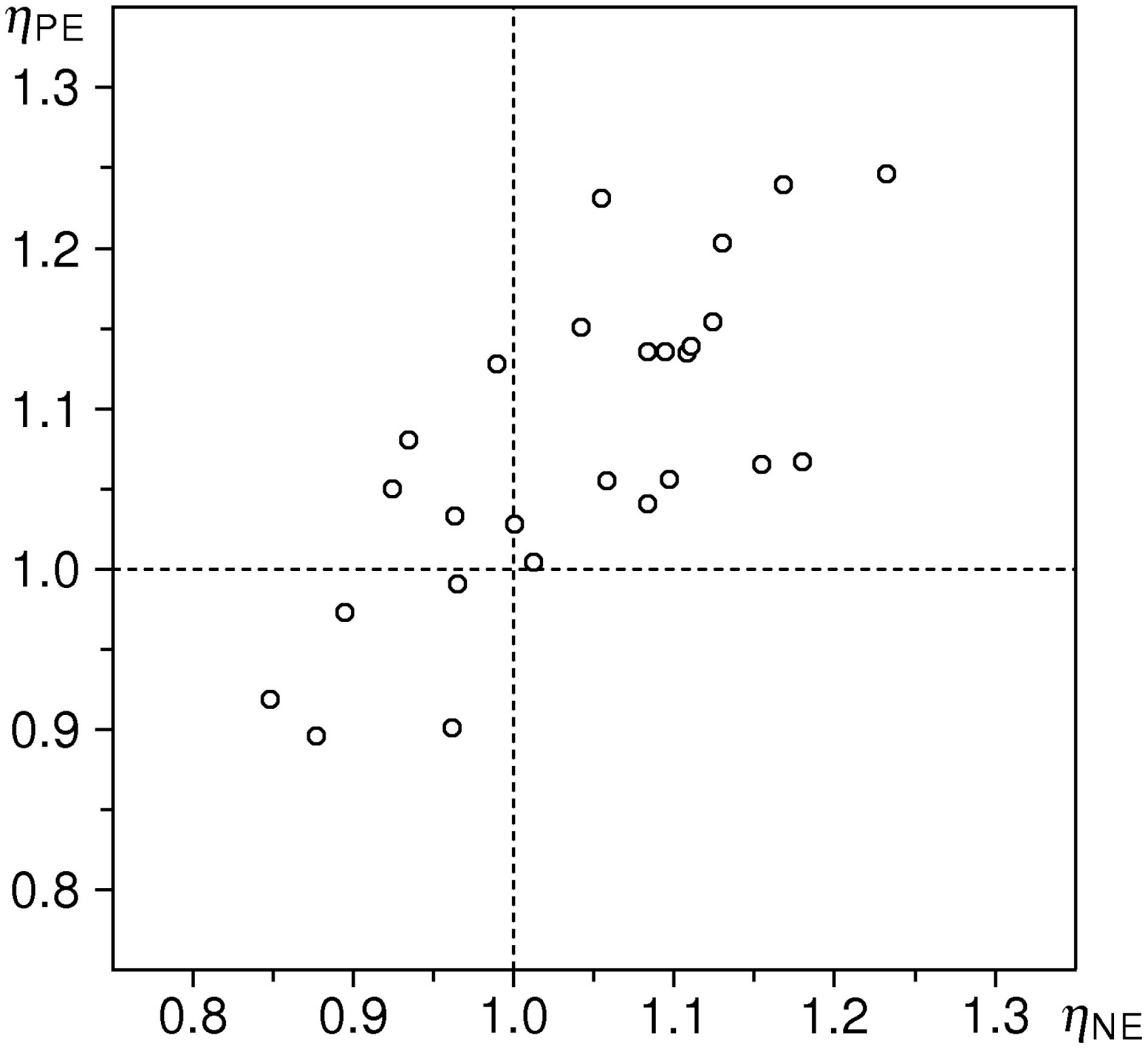
**Correlation plot of estimated flow ratios η_ne_ versus η_pe_ (*n* = 26, *R* = +0.744)**.

The main methodological innovation of the present study is the use of inflow ratios to quantify the stimulus-induced effect on duration perception. We wish to emphasize that our data-analytic approach makes these effects not only detectable—i.e., testable for their presence and statistical significance—but also measurable and comparable on a ratio scale. The quantification can work both ways: the method allows for characterization of stimuli of different kinds by their potence to alter perceived duration in a representative sample of observers. Or, inversely, susceptibility of individual observers to emotion-induced effects can be measured, using a standardized stimulus set, as in our study. Our data do not allow us to assess the intra-individual stability of those measures; repeated sessions and test–retest analyses would be required for that purpose. However, the notion of susceptibility to emotion-induced alteration of time experience, considered as individual trait, is supported by high intra-individual correlations between measures obtained separately for P and N stimuli: Pearson's correlation between *a*_n_ and *a*_p_ is *R* = +0.566 (*df* = 29, *P* < 0.01, see Figure 5), and notably higher, *R* = +0.744, between η_ne_ and η_pe_ (*df* = 24, *P* < 0.001, see Figure 6). This higher intra-individual correlation obtained with the dkm-based parameters is an additional argument in favor of our model-based approach^5^.

Another interesting, even if only qualitative, observation concerns the distribution of η_ne_ ratios (Figure 4). In fact, the local maximum at η_ne_ ≈ 1.1 almost exactly coïncides with that for η_pe_, and the sample mean of η_ne_ = 1.04 thus somewhat underestimates the effect of negative-valence stimuli. This is due to a secondary protrusion in the η_ne_ distribution, seen at values slightly below 1. The shape of the distribution thus suggests a mixture of two unimodal distributions, that of “responders” of the same average level as to the positive-valence stimuli, and that of “non-responders” to negative-valence stimuli. A deconvolution of the η_ne_ distribution would be interesting, but such analysis is not possible because of a rather small sample size.

Concluding, the present study (1) confirmed and extended evidence for emotion-induced alterations of duration perception, (2) demonstrated the practical utility of the dual klepsydra model for quantification of such effects, and (3) corroborated the hypothesis of internal time representation as resulting from integration of flows of interoceptive signals reflecting emotionally modulated bodily states. Saying that our approach opens a “window on” neurophysiology of subjective time might be unjustly exaggerated; integration of behavioral response measures with simultaneous physiological measurements would be desirable. It is not exaggerated to say, however, that the quantitative methods designed for the purpose of the present study open at least a “peephole” into the integration mechanisms underlying duration representation, and subjective time awareness in general.

## Acknowledgments

Marc Wittmann was supported by the European project COST ISCH Action TD0904 “Time In MEntaL activitY: theoretical, behavioral, bioimaging and clinical perspectives” (TIMELY; *http://www.timely-cost.eu*). We wish to thank Oksana Gutina for conducting part of the experiments and for general assistance. We are thankful to two reviewers of the original manuscript for critical remarks and constructive suggestions.

### Conflict of interest statement

The authors declare that the research was conducted in the absence of any commercial or financial relationships that could be construed as a potential conflict of interest.

## Figures and Tables

**Figure A1:**
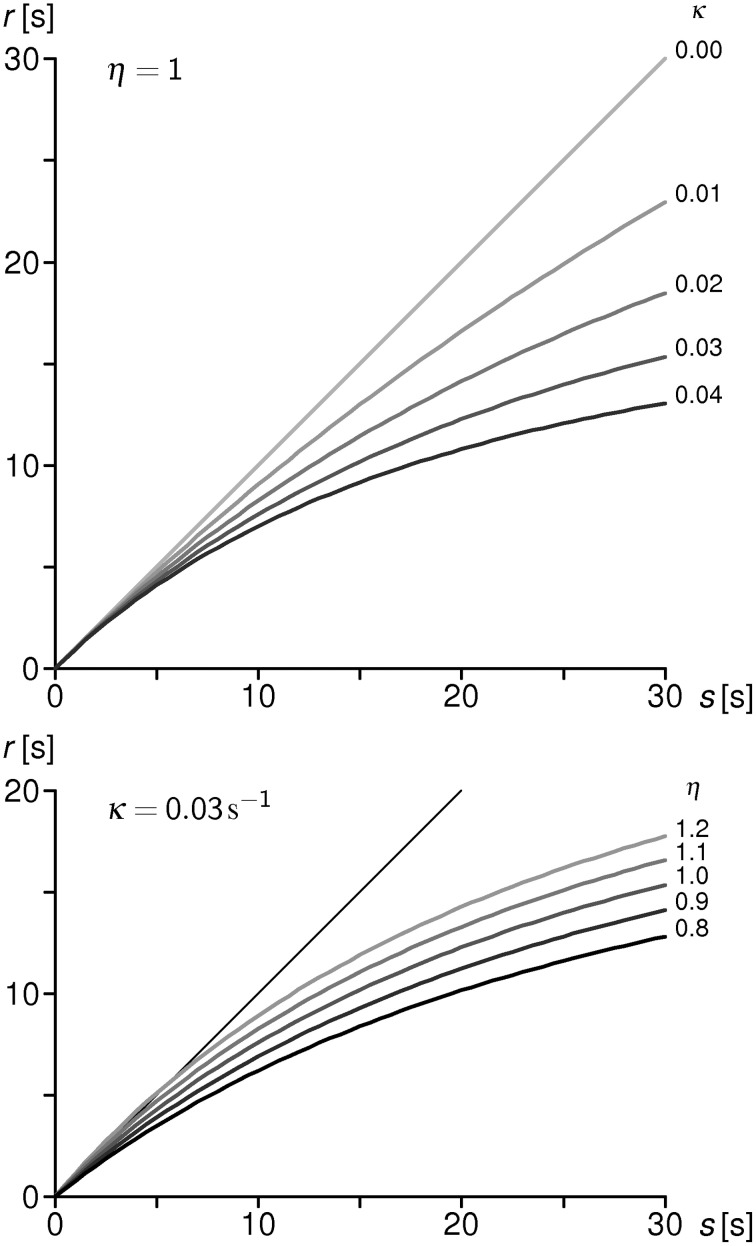
**Upper part:** different forms of the krf for η = 1 (equal inflows) and κ varied from 0 to 0.04 with step 0.01. The higher the loss coefficient κ, the more pronounced is the krf curvature, i.e., the progressive shortening with increasing duration *s*. **Lower part**: different forms of the krf for a constant value of κ = 0.03 and η varied from 0.8 to 1.2 with step 0.1. The curvature is basically determined by κ and further modulated by the inflow ratio η.—ISI in both displays are constant 0.5 s. Thin diagonal line indicates the chronometrically correct response *r* = *s*.

## Footnotes

^1^Obviously, this is a fair test as long as differences *r_v,i_* − *r*_e_,*i* are relatively small. However, for larger differences we may have a “built in” asymmetry in the evaluation procedure, since negative differences are mapped to the finite domain (0,1) while positive differences are mapped to the infinite domain (1,∞). To symmetrize the distribution, we apply log-transformation to the *a_v_* values and run a *t*-test on log *a_v_* against the expected value of 0.

^2^The *t*-tests applied to log-transformed indices yield *t* = 0.951 (ns) for the condition N, and *t* = 4.582 (*P* < 0.001) for the condition P, thus confirming the findings based on *a_v_* indices.

^3^ The *t*-tests applied to log-transformed inflow ratios yield *t* = 1.905 (*P* ≈ 0.06) for η_ne_, and *t* = 3.958 (*P* < 0.001) for η_pe_. In spite of somewhat reduced significances, the results are in agreement with those obtained for original inflow ratios.

^4^To substantiate this claim, we calculated statistics of the aggregated ratios after removing the five subjects we had excluded from the dkm analysis: mean *a*_n_ = 1.0169, mean *a*_p_ = 1.0758. One-sample *t*-tests against 1 yield *t* = 1.024 (ns) for the condition N and *t* = 4.720 (*P* < 0.001) for the condition N (*df* = 25 for both tests), differing only minimally from those obtained for the whole sample of 31 subjects (**section 4.2**). Since these results are based on the same subset of 26 subjects that entered the dkm analysis, we conclude that the better performance of the dkm-based strategy is not an artifact due to the difference between the two samples.

^5^ After removing the five subjects that were excluded from the dkm analysis, the correlation between *a*_n_ and *a*_p_ becomes slightly insignificantly higher: *R* = +0.589 (*df* = 24, *P* < 0.01), still clearly below the intra-individual correlation between the dkm-based flow-ratio estimates.

## Appendix

### A dual klepsydra model: the essentials

A linear inflow–outflow unit (iou) is described by an ordinary differential equation

(A1) $$\frac{\text{d}y}{\text{d}t}=i-\kappa y,$$

where *y* denotes the momentary state, *i* is the “inflow” intensity, and κ > 0 is a constant. For *i* being a piecewise constant function of time *t*, the iou state *y* is a piecewise exponential function. The dual klepsydra model (dkm), designed specifically for the duration reproduction paradigm, consists of two such ious (Figure 1). During the encoding phase (0 ≤ *t* ≤ *s*), iou_1_ is filled with intensity *i*_1_ > 0, and *i*_1_ = 0 afterwards. During the reproduction phase (*s*+ *w* ≤ *t*), iou_2_ is filled with intensity *i*_2_ > 0, until the equality of states, *y*_1_(*t*) = *y*_2_(*t*) is reached and the subject's response elicited at time *t* = *s* + *w* + *r*. The following relation holds for durations *r*, *s* and *w*:

(A2) $$\frac{{\text{e}}^{\kappa r}\text{​}-1}{1-{\text{e}}^{-\kappa s}}=\frac{i_{1}}{i_{2}}{\text{e}}^{-\kappa w}.$$

Denoting, for short, η ≡ *i*_1_/*i*_2_, and rewriting Equation (A2) to express the reproduced duration *r* as a function of the encoded duration *s* and the pause *w* between the two intervals, we obtain

(A3) $$r=\kappa^{-1}\ln\left(1+\eta \left(1-{\text{e}}^{-\kappa s}\right){\text{e}}^{-\kappa w}\right),$$

known as the “klepsydraic reproduction function” (krf). Its form is determined by two system parameters, κ and η, and an experimental parameter *w*. The krf is a monotonically increasing, negatively accelerated function of *s*, approaching asymptotically a limiting value for *s*→∞. Parameter κ measures the intrinsic loss of the hypothetical neural integrators; its physical dimension is [κ] = s^−1^. The inverse value κ^−1^ is proportional to the half-outflow time of the iou with zero inflow. The larger is κ (i.e., the shorter the characteristic time κ^−1^), the stronger is the negative curvature of the krf, and the more pronounced is the progressive shortening effect in the reproduction data (Figure A1).

In experiments employing stimuli of the same physical or psychological characteristics for the encoding and reproduction phase, η = 1 is usually assumed. A krf fit to reproduction data, using e.g., the weighted least squares method (Wackermann and Ehm, 2006), yields an estimate of the parameter κ. Reciprocally, if κ is known, and experimental conditions are varied between the encoding and reproduction phase, the corresponding inflow ratio η between conditions can be calculated (Wackermann and Meyer-Blankenburg, 2009). Simultaneous fitting of κ and η to a single data-set is not recommendable: estimation errors for κ and η, respectively, are strongly correlated (Wackermann and Ehm, 2006) and simultaneous estimates of the two parameters are thus unreliable.

For κ → 0 (loss-less integration) the krf becomes a linear function of *s*, *r* = η s, that is, a deviation from veridical reproduction can only be assessed in terms of flow ratios. In that case the dkm is functionally equivalent to the pcm.

### B rationale for the aggregate ratios analysis

Tests for effects of emotional valence of presented stimulus w.r.t. the neutral condition E can be based on differences or ratios between average responses. For our purpose the use of ratios *r_v_*/*r*_e_ is preferable, as these ratios are naturally linked to the “pacemaker-counter” model (pcm) (Zakay and Block, 1997).

Let *f*_1,*v*_ denote the pulse frequency during encoding of the first interval, *s*, in a condition *v*. The ratio between the reproduced duration *r* and the encoded duration *s* then gives an estimate of the inverse ratio between the respective frequencies, *r_v_*/*s* = *f*_1,*v*_/*f*_2_. The second stimulus used in the reproduction phase being always the same, we assume a constant pulse frequency *f*_2_ in both conditions.

Thus we have

(B1) $$\frac{r_{v}}{r_{\text{E}}}=\frac{r_{v}/s}{r_{\text{E}}/s}=\frac{f_{1,v}/f_{2}}{f_{1,\text{E}}/f_{2}}=\frac{f_{1,v}}{f_{1,\text{E}}}$$

This means that the between-conditions ratio of the reproduced durations provides a direct estimate of the between-conditions ratio of the pulse frequencies. The aggregate indices *a_v_* (Equation 2) used in our analyses provide estimates of the respective frequency ratios averaged across all durations *s_i_* (*i* = 1,2,3).

### C rationale for the DKM-based analysis

Our analysis was based on the hypothesis that emotional valence of presented acoustic stimuli modulates the inflows entering the neural integrators. Emotional valence was manipulated during the encoding phase; assume that for an individual subject *i*_1_ attains three different values, *i*_p_, *i*_n_, or *i*_e_, depending on the stimulus. The second stimulus used in the reproduction phase was always the same, a sine-wave reference tone, so we assume that the inflow intensity *i*_2_ is constantly *i*_ref_. The inflows *per se* are inobservable; only data-based inferences on inflow ratios are possible. For the three emotion conditions we define

(C1) $$\eta_{\text{p}}\equiv \frac{i_{\text{p}}}{i_{\text{ref}}},\;\eta_{\text{N}}\equiv \frac{i_{\text{N}}}{i_{\text{ref}}},\;\eta_{\text{E}}\equiv \frac{i_{\text{E}}}{i_{\text{ref}}}.$$

Of interest now are effects of emotionally laden stimuli, P or N, w.r.t. the neutral stimulus E. For this purpose we take ratios

(C2) $$\eta_{\text{PE}}\equiv \frac{\eta_{\text{P}}}{\eta_{\text{E}}}=\frac{i_{\text{P}}}{i_{\text{E}}},\;\eta_{\text{NE}}\equiv \frac{\eta_{\text{N}}}{\eta_{\text{E}}}=\frac{i_{\text{N}}}{i_{\text{E}}},$$

providing net inflow ratios between emotion versus neutral conditions.

A prior knowledge of individual κ is needed to estimate η_*v*_ for any of the three conditions *v* = P, N, E. Ideally, κ should be estimated from a data-set with perfectly homogeneous stimuli (e.g., reference tone used for both encoding and reproduction), but our experimental design does not yield such data. As an heuristic approximation we estimate κ from a merged data-set, i.e., not distinguishing between emotion conditions and putting formally η = 1. This is not quite correct, because 2/3 of the data were obtained for emotion-versus-reference stimuli, and so the subsequent estimates of η_*v*_ (Equation C1) may be somewhat biased. However, we are mainly interested in η_*v*E_ ratios: as seen from Equation (C2), the unknown intensity *i*_ref_ cancels out, and it is thus legitimate to expect that the procedural bias is compensated by this operation.

For κ → 0 (loss-less integration) the ratio *r_v_*/*s* gives a direct estimate of η_*v*_, by analogy to the pcm (cf. Equations B1, C2).
